# Factors associated with induced abortions in Pakistan: a comprehensive analysis of Pakistan maternal mortality survey 2019

**DOI:** 10.3389/frph.2025.1536582

**Published:** 2025-07-10

**Authors:** Samina Naeem Khalid, Farid Midhet, Qudsia Uzma, Ellen Mpangananji Thom, Shehla Baqai, Muhammad Talha Khan, Areeba Memon

**Affiliations:** ^1^ MNCH Department, Health Services Academy, Islamabad, Pakistan; ^2^ Bahria University Medical & Dental College, Bahria Medical University Campus, Karachi, Pakistan; ^3^ MNCAH (Maternal Neonatal, Child and Adolescent Health), World Health Organization, NIH, Islamabad, Pakistan; ^4^ Reproductive Health, World Health Organization, NIH, Islamabad, Pakistan; ^5^ Resident Internal Medicine, SUNY Upstate Medical University, Syracuse, NY, United States

**Keywords:** abortions, maternal health, PMMS 2019, risk factors, pregnancy loss

## Abstract

**Background:**

Induced abortions (IA) remain a serious public health issue in Pakistan despite social constraints and legal prohibitions. The number is alarmingly high, and the study done by the Population Council (2012) indirectly estimated 2.2 million abortions per year and an abortion rate of 50 per 1,000 women.

**Methodology:**

This study reports the results of a secondary data analysis of the Pakistan Maternal Mortality Survey 2019 to compare the women who reported having an IA in the last three years with those having a live birth in the same period. A nested case-control comparison of women reporting IA as cases and those having a live birth as controls. Logistic regression was used to estimate odds ratios (OR) for major risk factors of maternal mortality after adjusting for women's age, parity, education, and wealth quintile.

**Results:**

Results show that recent use of family planning, having a prior history of pregnancy loss, and higher gravidity are all linked to IA (*P* < 0.05). On the other hand, neither the average household education nor the women's education affects the rates. The other associated factors include parity, past use of family planning, socioeconomic status, place of residence, and the educational level of women. These correlations are based only on uncorrected odds ratios and do not account for confounding variables.

**Conclusion:**

Women having past miscarriages, having several children, or improperly using family planning methods are more likely to have induced abortions. These findings can help medical professionals develop evidence-based policies and regulations.

## Background

Globally, an estimated 73 million induced abortions occur each year, roughly 29% of all pregnancies. Among the unintended pregnancies, 61% end up in an abortion, and most of these abortions are clandestine and unsafe (1). It is a major preventable cause of maternal mortality and morbidity. The complications of unsafe abortions lead to both physical and mental health issues, which place women in cumbersome social and financial affliction, and burden communities and the health care system. The lack of provision of safe, timely, affordable, and respectful post-abortion care services is a critical public health problem (2). Between 2010 and 2014, Ganatra and colleagues estimated that 25 million unsafe abortions were performed worldwide, while a staggering 88% occurred in developing countries. Asia, in particular South and Central Asia, contributed to more than half of these unsafe abortions. This sheds light on the impact of unsafe abortion in places with already limited access to services of safe abortion (3).

Despite a significant decline in fertility since the 1990s, a relatively high total fertility rate (TFR) grapples Pakistan with a current estimate of 3.6 births per woman. The unmet need for family planning remains high at about 17.3%, and the majority of pregnancies are unintended (4). The most likely reason for this is the low use of modern contraceptives: just 48.6% of couples utilize a modern method of family planning. In Pakistan, the only data on the incidence of induced abortions comes from indirect studies. Using the Abortion Incidence Complications Method (AICM), the Population Council estimated that the number of induced abortions was 2.25 million in 2012, with an abortion rate of 50 per 1,000 reproductive-age women (15–49 years) (5). In a second round of this study in 2023, the abortion rate was estimated at 66 per 1,000 women of reproductive age (6). Post-abortion complications were reported to have increased in this period, from 15% in 2012 to 21% in 2023.

The AICM mainly uses data on women undergoing post-abortion care (PAC) reported in health facilities to indirectly estimate the total incidence of induced abortions. To account for abortions that do not require medical care, the AICM uses a multiplier, which is the inverse of the fraction of abortions that result in the utilization of facility-based care. Though the AICM attempts to distinguish between induced and spontaneous abortions, it assumes an implausibly low (indeed, factually incorrect) incidence of miscarriages. This methodological flaw therefore concludes an artificially or erroneously high number of induced abortions. Moreover, the AICM assumes that miscarriages rarely require medical care, even though they occur in 10%–20% of pregnancies and many need treatment. In Mexico, AICM estimates were up to 10 times higher than actual figures due to misclassified miscarriages (7). Critics argue that reliance on aggregated health facility data further distorts results, especially in regions with limited diagnostic capabilities.

Pakistan's maternal mortality ratio (MMR) declined from 276 deaths per 100,000 live births in 2007 (Pakistan Demographic and Health Survey) to 186 in 2019 (Pakistan Maternal Mortality Survey). During this period, the proportion of maternal deaths due to abortion-related complications doubled, from 5% in 2007 to 10% in 2019. However, it may be noted that the assignment of cause of maternal death in the two surveys was through a process of verbal autopsy (VA) interviews and a review of VA information by a panel of experts and that the classification of causes may not always explicitly distinguish induced abortion from spontaneous abortion (miscarriage) or ectopic pregnancy (8).

Abortion has remained a controversial subject in Pakistan, which practices a near-total ban on this procedure. More recently, the government of Pakistan has begun to shoulder more responsibilities to address safe abortion service provision within the current laws, together with NGO support (9). There remains, however, the practice of unsafe abortion, particularly in women of low socio-economic status, which leads to medical complications and death (10). Women in Pakistan are bound in an intricate knot of laws regarding abortion, where women have to bear mental, psychological, social, and legal consequences whenever they seek abortion services (11).

Abortion stigma still exists significantly in Pakistani society, which affects women who have had an abortion. This stigma often leads to secrecy, suffering, and isolation among the affected (12). Although the majority of Pakistanis appear to condone having an abortion as long as there is danger posed to physical health, this is far less accepted when the intended reason for abortion is mental health (13). Recently, there has been progress in the political scenario where both the NGOs and the government have worked together to focus on unsafe abortion as a public health issue (9). Additionally, measuring abortion prevalence in Pakistan presents considerable challenges due to the interlinking of legal restrictions and societal stigma. A survey in Karachi found that 16% of women over the age of 18 years had an abortion, much more than the rate of abortion obtained by asking respondents directly. This result indicates that indirect exposure may be successful in increasing the reporting of abortion (14).

Even though unsafe abortion is prevalent, as shown by the Pakistan Maternal Mortality Survey conducted in 2019 and other studies, there is still a lack of understanding of the underlying causes of induced abortion. This in-depth analysis, therefore, focuses on providing answers to the questions raised above in terms of the demographic, reproductive, and socio-cultural factors that influence abortion decision-making.

## Methodology

Pakistan Maternal Mortality Survey 2019 (PMMS 2019) was a national survey exclusively on maternal mortality and morbidity, which was conducted by the National Institute of Population Studies (4), Government of Pakistan, with technical assistance from the Demographic and Health Surveys (DHS), USAID. The sample included 136,226 households in the four provinces, AJK, and GB. Births and deaths of the last three years were recorded to identify maternal deaths and estimate the maternal mortality ratio. In a 10% sub-sample of households, 14,703 ever-married women aged 15–49 years were interviewed to identify complications of health services utilization in pregnancy, delivery, and postpartum in the last three years (8).

All women reporting a pregnancy in the last three years were asked about induced abortion; however, there was possible under-reporting (abortion rate was 12/1,000 women aged 15–49), and direct estimation of the abortion rates by biological, demographic, and socioeconomic factors would have been misleading. To overcome this difficulty, an unmatched nested case-control study was designed, whereby the women who reported an induced abortion in the last 3 years were regarded as **cases** (n_1_ = 170; sampling fraction = 1.00). The **controls** were randomly selected from women who reported having a live birth during the last three years (n_2_ = 1,729; sampling fraction = 0.20). Binary logistic regression (SPSS version 19.0) was used to estimate adjusted odds ratios (AOR) for the association between reported induced abortion and the risk factors of interest. The sampling scheme of the PMMS 2019 was such that the clusters were numerous (*n* = 1,096) and of very small size. Because the intra-cluster correlations were close to zero, the need for generalized estimating equations (GEE) was not critical. A comparison was made of the women reporting an induced abortion with those having a live birth in the same period to find the differences in biological and socio-demographic risk factors. The adjusted odds ratios, along with the 95% confidence intervals and *p*-value, were calculated.

## Results

The causal links of age, parity, and pregnancy history on the odds of induced abortion were adjusted for household socioeconomic status (14) Urban/Rural residences were analyzed. Table 1 presents the adjusted odds ratios (AOR) and 95% confidence intervals (CI) to assess the significance of the associations. The history of prior pregnancy loss indicates that women with a history of at least one pregnancy loss have 1.55 times higher odds of induced abortion compared to those with no previous pregnancy loss (*p* < 0.05). Table 1 further shows that the odds of induced abortion do not significantly differ between nulligravida (no previous pregnancies) and women with 1–5 pregnancies. However, women with six or more pregnancies have 2.65 times higher odds of induced abortion compared to nulligravida (*p* < 0.05).

**Table 1:** Association of history of pregnancy loss, gravidity, ever use of family planning, women's education, and household average education on induced abortions: adjusted odds ratios (AOR) and 95% confidence limits (adjusted for age, parity, household's SES, and urban/rural residence).

Variable	Categories	AOR	95% confidence interval	*P*-value
History of pregnancy loss	None (Ref.)	–	–	
	≥1	1.55	1.10–2.18	0.012
Gravida* (number of pregnancies before the last pregnancy)	Nulligravida	0.91	0.53–1.57	0.732
	1–5 pregnancies (Ref.)	–	–	–
	6 + pregnancies	2.65	1.67–4.20	<0.001
Ever used family planning in the past	None (Ref.)	–	–	
	Modern	1.82	1.25–2.64	0.002
	Traditional	2.59	1.55–4.34	<0.001
Women's education	0–9 classes (Ref.)	–	–	–
	10 + classes	1.07	0.72–1.58	0.738
Household average education (of those ≥15 years)	0–9 classes (Ref.)	–	–	
	10 + classes	1.28	0.83–1.97	0.271

Women who have ever used modern or traditional family planning methods have significantly higher odds of induced abortion compared to those who have not used any family planning methods. The odds ratios are 1.82 and 2.59, respectively. There is no significant association between a woman's education level (10 or more classes) and the odds of induced abortion. Also, there is no significant association between the household's average education level (10 or more classes) and the odds of induced abortion.

Table 2 presents the unadjusted OR and 95% CI for the association of age, parity, and pregnancy history with the odds of induced abortion. In terms of age group, compared to women aged 25–34 years (reference category), those under 25 years had slightly lower odds of induced abortion (OR = 0.69). Still, this difference was not statistically significant (*p* = 0.072). Similarly, women aged 35 years and above had slightly higher odds of induced abortion (OR = 1.31), but again, this difference was not statistically significant (*p* = 0.531).

**Table 2:** Association of age, parity, and pregnancy history on the odds of induced abortion: unadjusted odds ratios and 95% confidence limits.

Variable	Categories	Odds ratio	95% confidence interval	*P*-value
Age group	<25 years	0.69	0.46–1.03	0.072
	25–34 years (Ref.)	–	–	–
	≥35 years	1.31	0.77–1.66	0.531
Parity	live births	2.87	1.95–4.22	<0.001
	2–4 live birth (Ref.)	–	–	
	≥5 live births	1.57	1.07- 2.30	0.021
History of prior pregnancies lost	None (Ref.)		–	–
	≥1	1.55	1.12–2.15	0.008
Gravida (number of pregnancies before the last pregnancy)	Nulligravida	0.92	0.55–1.56	0.780
	1–5 pregnancies (Ref.)	–	–	–
	6+ pregnancies	1.87	1.28–2.71	<0.001
Ever used family planning in the past	None (Ref.)	–	–	
	Modern	1.80	1.28–2.54	0.001
	Traditional	2.82	1.73–4.61	<0.001
Household SES (wealth quintile)	Lowest (Q-1) (Ref.)	–	–	
	Middle (Q-2 to Q-4)	4.68	2.43–9.00	<0.001
	High (Q-5)	6.35	3.11–12.97	<0.001
Residence	Rural (Ref.)	–	–	–
	Urban	1.67	1.22–2.29	0.001
Women's education	0–9 classes (Ref.)	–	–	–
	10+ classes	1.66	1.18–2.32	0.003
HH average education (of those ≥15 years)	0–9 classes (Ref.)	–	–	
	10+ classes	1.97	1.34–2.89	0.001

Regarding parity, women with 2–4 live births (reference category) had significantly higher odds of induced abortion compared to those with 1 live birth (OR = 2.87, *p* < 0.001). On the other hand, women with five or more live births also had slightly higher odds of induced abortion (OR = 1.57, *p*-value of 0.021). Considering the history of pregnancy loss, women with at least one pregnancy loss had significantly higher odds of induced abortion compared to those with no history of pregnancy loss (OR = 1.55, *p* = 0.008).

Table 2 also shows that the nulligravida women (those who have never been pregnant before) had slightly lower odds of induced abortion compared to women with 1–5 pregnancies (OR = 0.92), not statistically significant (*p* = 0.780). However, women with 6 or more pregnancies had significantly higher odds of induced abortion (OR = 1.87, *p* < 0.001).

When considering the use of family planning methods in the past, or having ever used modern methods of family planning, there were significantly higher odds of induced abortion compared to those who had not used any family planning methods (OR = 1.80, *p* = 0.001). Similarly, women who had used traditional family planning methods had even higher odds of induced abortion (OR = 2.82, *p* < 0.001).

Table 2 also shows that compared to the women in the lowest SES category (Q-1), middle (Q-2 to Q-4), and high (Q-5) SES categories had significantly higher odds of induced abortion (OR = 4.68 and 6.35, respectively, both *p* < 0.001). Similarly, women residing in urban areas had significantly higher odds of induced abortion (OR = 1.67, *p* = 0.001). Additionally, women with higher levels of education had significantly higher odds of induced abortion (ORs = 1.66, *p* < 0.001). Similarly, in households with higher average education, the odds of induced abortion were also significantly higher (OR = 1.97, *p* < 0.001).

According to Table 3, the use of long-acting Reversible Contraception (LARC) before the last pregnancy was associated with significantly higher odds of induced abortion (OR = 2.75, *p* = 0.002). Similarly, the use of traditional family planning methods was also associated with significantly higher odds of induced abortion (OR = 2.62, *p* < 0.001). On the other hand, the use of short-term family planning methods was associated with moderately higher odds of induced abortion (OR = 1.68, *p* = 0.011). The use of modern family planning methods also showed a significant association with increased odds of induced abortion (OR = 1.82, *p* = 0.002).

**Table 3:** Family planning method ever used (before the last pregnancy) and the odds of induced abortion.

Family planning methods	Odds ratio	95% confidence interval	*P*-value
LARC	2.75	1.43–5.29	0.002
Short term	1.68	1.13–2.49	0.011
Traditional	2.62	1.56–4.39	<0.001
Modern	1.82	1.25–2.64	0.002

The odds of Induced Abortion with sons and daughters alive show that if there are no previous children (reference group), women with 1–2 sons alive had significantly higher odds of induced abortions (OR = 1.90, *p* = 0.013). However, women with three or more sons alive did not show a significant difference (OR = 0.88, 95% CI: 0.57–1.37, *p* = 0.584). Similarly, women with 1–2 daughters alive had significantly higher odds of induced abortions compared to women with no previous children (OR = 2.03, *p* = 0.007). On the other hand, women with three or more daughters alive did not show a significant difference in the odds of induced abortions (OR = 1.02, *p* = 0.924).

In the present analysis, however, no significant differences in self-reported illness before or during pregnancy were found between women having an abortion and those having a live birth, except for gestational diabetes, which was significantly higher among women who had a live birth. Similarly, there were no significant differences in receiving antenatal care from a skilled provider between the two groups. The PMMS 2019 was a household survey, and data on fetal anomalies were not available. Also, there were no significant differences in the health-seeking behavior of pregnant women having a live birth or having an induced abortion. Unfortunately, PMMS 2019 did not elicit information about the reasons for the decision to seek an induced abortion.

## Discussion

The analysis of the PMMS 2019 offers an account of various factors that influence maternal mortality in the country (8). This article aims to provide a comprehensive overview of induced abortions based on the findings of this survey. The goal is to contribute to the existing body of knowledge and guide efforts to improve women's reproductive health outcomes in Pakistan. According to the data from the Guttmacher Institute, Pakistan performed almost 2.2 million unsafe abortions in 2012 (5). This emphasizes how urgently more research is required to explore the causes and policy changes to address the issue.

The subject of unsafe abortion is quite complicated and is impacted not only by medical, health, and related variables but also by social and legal challenges. This study highlights the intricate connections between variables influencing induced abortion by looking at the causal links of age, parity, pregnancy history, family planning use, and socioeconomic factors.

The issue of lack of prenatal care and delayed recognition of an unwanted pregnancy are potential contributors to the decision to seek an induced abortion. The data of the present study showed the limitation that women do not seek medical care early in the first trimester of pregnancy, leading to a lack of identification of the complications or abnormalities. This delay can sometimes lead to more complex decisions about abortion later in pregnancy, where options may be more limited. In the present analysis, however, no significant differences were found in health-seeking behavior between the pregnant women who had a live birth and those who had an induced abortion. Utilization of antenatal care does not seem to have an association with the induced abortions reported in the PMMS 2019.

This study showed that age may not significantly impact the likelihood of induced abortions within the studied population. However, age has frequently been mentioned in previous research as a possible contributing factor because of its link to higher parity or unwanted pregnancies (15–17). The absence of notable results in this particular study, however, suggests that other factors might have a greater influence on abortion decisions than age.

There is a significant link between parity and induced abortion. If a woman has two to four live births instead of one, her chance of having an induced abortion is much lower. This could be because women who have had more children tend to be more stable and have more knowledge of reproductive health choices. On the other hand, an increased, albeit not statistically significant, odds of induced abortion are shown in women who have had five or more live births. Socioeconomic pressures related to increased family numbers or health issues could cause this trend. Women who have had more live births are more likely to choose an induced abortion, suggesting that some interventions are required for women who may find it difficult to use family planning (FP). Multiple other studies done in various countries have shown rates of increased induced abortion with increasing parity (18–20).

The findings also suggest that the odds of induced abortion are greatly increased by the history of a miscarriage. Women who have lost a pregnancy at least once are 1.55 times more likely to have an induced abortion than women who have never lost a pregnancy before. This result emphasizes how crucial it is to take prior pregnancy experiences into account when assessing the odds of abortion, as they might be affected by psychological or medical issues that influence their choices for future pregnancies (21–23).

The analysis of the present study shows higher odds of induced abortion with ever use of family planning (FP) methods. This apparent paradox may reflect the influence of reproductive intention, where women using contraception are more motivated to avoid pregnancy and are thus more likely to terminate an unintended pregnancy if it occurs. Moreover, the phenomenon of risk compensation may play a role, wherein the perceived protection provided by FP may incentivize increased sexual activity, thus raising the chances of failure-related unintended pregnancies. In addition, the predominance of short-term or traditional contraceptive methods with higher failure rates may contribute to this trend. These findings underscore the importance of improving access to, and education about, effective contraceptive use and reproductive autonomy (24, 25).

Other factors observed in this study linked with induced abortion include: education levels, urban areas, and socioeconomic status (18). There were higher odds of induced abortion among women who have high socioeconomic status, live in cities, and have more education. Higher socioeconomic status (SES) individuals often face greater opportunity costs, such as disruptions to their career progression, higher education, or modern lifestyle, which can have an impact on their reproductive choices. As a result, they may be more likely to seek an abortion when facing an unintended pregnancy. This pattern might result from complex relationships involving cultural factors, reproductive health awareness, and healthcare availability. Greater access to family planning services may also result from higher SES and education levels, but there may also be more pressure or expectations impacting abortion decisions. The findings of the present study are contrary to the study done in Ethiopia, which shows that illiteracy leads to a lack of uptake of family planning services, leading to unwanted pregnancies (26). Other studies also show that family planning services help to reduce induced abortion rates (27, 28). A study done in Bali also showed these factors influencing rates of induced abortion (28).

According to a study done by the Guttmacher Institute, if all unmet requirements of FP are addressed, the cost of abortion care could be drastically decreased because contraception reduces unplanned pregnancies. For every dollar more spent on increasing access to contraception, Pakistan would save $ 4.52 on the cost of maternity, neonatal, and abortion care for all women of reproductive age (29).

Pakistan's Penal Code of the 1860 legal framework only authorizes abortions when necessary to save a woman's life or for medical purposes. This poses consequences of the refusal of doctors to do abortions on demand and leads to the use of unskilled force, such as Dais (Traditional Birth Attendants) and Quacks, by women seeking induced abortions, risking their lives. Couples also face anxiety about being judged by society, nervousness about judging themselves, and silence among women who have undergone abortion procedures (11). It also inhibits access to post-abortion care (PAC) services, which is important in the reduction of maternal mortality caused by unwanted pregnancies (30). Neighboring countries like India have very high unsafe abortion rates even after having legalized abortion through the 1971 Medical Termination of Pregnancy Act (MTPA). A study published in 2019 showed an incidence of 67% unsafe abortions when abortion had been legalized for around 50 years (31). Moreover, evidence suggests that while abortion was responsible for only 8% of maternal deaths before 1972 in India, that proportion increased to 15%–30% after 1972 (32). This raises doubts about whether restrictive abortion laws cause more unsafe abortions, from the closest country for which we have such evidence. There is a need to broaden the availability of contraceptives, provide better training for providers, and include men in family planning programs (33).

The present study draws attention to the complex relationship between demographic, reproductive, and socioeconomic factors and induced abortion in Pakistan. Reproductive health education should be prioritized, and access to high-quality family planning services should be enhanced through targeted measures. Programs specifically designed to account for distinct sociocultural backgrounds can close gaps in contraceptive use efficacy.

It is crucial to remember that, due to legal limitations and the delicate nature of the subject, there is a dearth of information, and it is difficult to gather reliable data on the frequency and causal links of induced abortions, as many women choose to undergo the procedure in secret and under dangerous circumstances. This research can help close the information gaps and provide guidance for policies and interventions aimed at enhancing women's reproductive health outcomes. An extensive strategy is needed to devise and comprehend contextual elements to create culturally aware and efficacious interventions. In Pakistan, the Federal Ministry of National Health Services Regulations and Coordination, in collaboration with Ipas, has developed and endorsed national and provincial guidelines for the provision of post-abortion care (PAC) services. These guidelines aim to improve healthcare outcomes by ensuring that PAC procedures are carried out in a safe and medically sound manner (34, 35).

## Data Availability

The datasets presented in this study can be found in online repositories. The names of the repository/repositories and accession number(s) can be found in the article/Supplementary Material.
